# Terror-related injuries in Somalia: a retrospective cohort of 2426 hospitalized cases along 7 years

**DOI:** 10.1038/s41598-022-22276-z

**Published:** 2022-10-14

**Authors:** Ebubekir Arslan, Abdikarim Hussein Mohamed, Osman Cetinkaya

**Affiliations:** Mogadishu Somalia Turkish Training and Research Hospital, Mogadishu, 2526 Somalia

**Keywords:** Trauma, Epidemiology

## Abstract

Terrorism-related injuries and deaths have become a major threat to the Somalian population, as in the rest of the world. We aimed to characterize and compare firearm and explosion injuries caused by terrorist acts. This retrospective study reviewed the data of all patients injured by explosions and firearm attacks over seven years. Epidemiological characteristics, injury pattern, severity scores, hospital resource utilization parameters, length of stay, and death rates were evaluated. A total of 2426 patients were injured by 359 explosions and firearm attacks during the study period. Eighty-one percent of the patients (n = 1974) were male, while 19% of the cases were female. Multiple body site injuries occurred more frequently in explosion injuries (75%) than firearm wounds (48%) (*P* < 0.001). The relative frequency of internal injuries in explosion injuries was higher than in firearm wounds (46.7% vs. 36.2%). Patients injured due to the explosion have a greater rate of severe and critical injuries than those injured by firearms (30.2% vs. 21.2%, *P* < 0.001). About a quarter (24%) of the patients were hospitalized in the intensive care unit. The inpatient mortality rate was 11.6%. The findings of the study revealed that suicide bombings explosions are associated with multiple body site injuries, a greater rate of severe and critical internal injuries, and a higher mortality rate.

## Introduction

Somalia has suffered from a complex humanitarian emergency for over two decades^1^. Terrorism-related injury and deaths have become a major threat to the population in Somalia, as in the rest of the world. These patients are deprived of adequate medical attention and treatment due to the lack of a central government and the limited capacity of health facilities, leading to increased morbidity and mortality associated with terrorism-related injuries.

The most common explosion-related injury mechanisms associated with terrorism vary depending on the type of bomb, the materials in the explosion environment, the distance between the patient and the explosion center point, and the use of protective barriers^2,3^. Injuries caused by the explosion can occur in five different ways: primary injury caused by direct contact with high-pressure shock wave resulting from the explosion (most commonly tympanic membrane rupture and explosion lung), secondary injury caused by flying materials and shrapnel after the explosion, tertiary injury caused by throwing the patient as a result of the explosion, quaternary injuries (i.e., miscellaneous explosion -related injuries) encompass injuries caused by burns, collisions, falling masonry, buildings, beams, et cetera and quinary injuries caused by clinical consequences of "post-detonation environmental contaminants" including bacteria, radiation (i.e., "dirty bombs"), tissue reactions to fuel, metals, et cetera^2,3^. For these reasons, many patients affected by the explosion may experience life-threatening injuries affecting multiple systems.

With the increase in terrorist activities, physicians, especially emergency medicine doctors, have to improve their knowledge of terror-related injury mechanisms and their treatment skills for victims of mass injury events^4^. Mass casualties are often characterized by a quantity, severity, and diversity of injuries^5^.

Terror-related injuries, armed conflicts, and natural disasters are significant causes of fatalities and injuries worldwide, particularly in low-income countries with limited health facilities. Head, neck, and abdomen injuries are the most common cause of death with the worst prognosis. Over the last two decades, more than two million persons and more than 1.4 million have been lost through terrorist activities and armed conflicts. Somalia, Afghanistan, Ethiopia, Bangladesh, the Congo Republic, Indonesia, Iraq, Liberia, Pakistan, and Sudan are the countries most affected by the substantial mortality^6^.

Terrorist attacks in Somalia generally consist of two main forms: suicide bomber explosions and firearm injuries caused by conflicts. This is the first study to describe and compare the clinical profile, attack frequency over time, severity of related injuries, and hospital resource usage of explosions and firearm injuries caused by terrorist acts.

## Method

This retrospective cohort study included data from May 1, 2014, and April 30, 2021, using the Hospital Information System (HIS) electronic medical records. Mogadishu-Somali Turkish Training and Research Hospital is the only hospital in Somalia that offers tertiary healthcare (highly specialized medical care offering advanced and complex procedures and treatments in state-of-the-art facilities). Thus, it is the only trauma center.

### Data collection and processing

Terrorism-related injuries have been identified in records of ICD E-codes E979 and E990 to E999. For identified patients, terrorism-related firearms, explosions, and other injuries were defined according to specific ICD E-codes and a clear text description of the injury. Procedures comparing ICD E-codes and Abbreviated Injury Score diagnoses were performed. Each attack was defined according to date and mechanism, and after each terrorism-related attack, it was compared and controlled with the data in the "Social Events Information Notes" sent to the Ministry of Health. Internal injuries within the range of ICD-9-CM codes 850–854 and 860–868 were divided into three parts: head, trunk, and abdominal injury body regions.

The two injury mechanisms (i.e., firearm and explosion) were compared in terms of clinical parameters, including situational parameters, demographic characteristics, Abbreviated Injury Score, Injury Severity Score, injured body regions, total number of injured body regions, operating room and intensive care unit use, length of hospital stay, in-hospital mortality and time. Scoring methods such as Abbreviated Injury Scale (AIS8) and Injury Severity Score (ISS9) were used for injury severity classification^7–10^.

The Mogadishu Somali Turkish Training and Research Hospital Clinical Research Ethics Committee approved this study (approval number MSTH/6752). All methods were performed per the relevant guidelines and regulations. All patients previously consented to use their medical and surgical records for research purposes.

### Inclusion and exclusion criteria

All adult patients (i.e., > 18 years) with a sustained injury brought to the emergency department due to terrorism-related injuries were included in the study. Pediatric patients (i.e., < 18 years) were excluded from the study due to the different injury patterns, trauma management algorithms, trauma assessment score systems, and treatment modalities in this particular population. In addition, patients who were considered dead at the scene or on arrival at the hospital were excluded from the study.

### Definitions

Indicators of hospital resource use include the length of stay (LOS), intensive care treatment, and surgical procedures. The framework for the analysis of injury diagnoses was based on the Barell body region due to the nature of the injury diagnosis matrix. The matrix was modified to include five injury types: fractures, internal injuries, open wounds, burns, and others. In this matrix, nine body regions were defined as traumatic brain injury (TBI), other head injuries, spinal cord and column, chest, abdomen, pelvis, trunk, back and buttock, upper extremities, lower extremities, and others. "Number of injured patients/number of attack" ratio was used to to achieve injury rates.

### Statistical analysis

The data were analyzed using descriptive univariate analysis. For categorical variables, the frequencies and percentages were reported as point estimates. Quantitative variables, the mean ± (SD), were used whenever possible. Continuous variables were compared using Student's t-test, whereas categorical variables were compared using Pearson's chi-square (χ^2^) or Fisher's exact test. *P* value < 0.05 was considered statistically significant.

### Institutional review board statement

This study was approved by the Clinical Research Ethics Committee of the Mogadishu Somali Turkish Training and Research Hospital (approval number MSTH/6752). All methods were performed in accordance with the relevant guidelines and regulations.

### Consent to participate

Using the electronic medical records on the hospital information system and no harm to the patients; our institution’s Ethical Committee was waived the informed consent.

## Results

### Attack type and frequency over time

**Figure 1:**
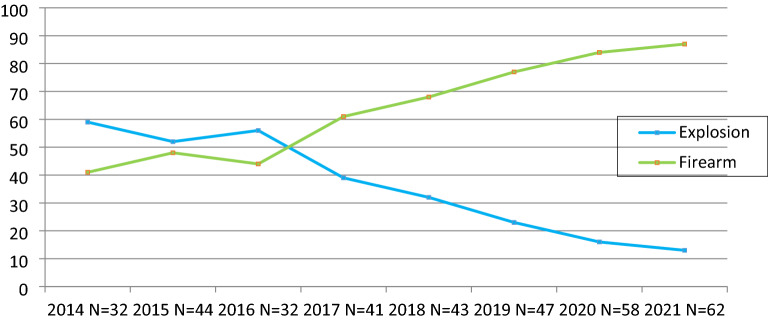
Variation of attack frequency over time by different terrorist mechanisms (number of attacks, not number of patients).

A total of 2426 patients were injured by 359 explosions and firearm attacks during the study period. Most patients were injured due to firearm attacks (N = 1353, 56%), while explosions injured 44% of patients (N = 1073). The attacks were primarily (n = 241, 67%) acts of firearm terrorism, while the remaining were explosions. The total number of attacks has increased over the years, mostly due to the increase in firearm wounds. Conversely, the relative frequency of explosion -related attacks and injuries decreased significantly over the years. Figure 1 shows the distribution of the frequency of attacks from two different terror mechanisms overtime during the study period. In the data we obtained, the temporal distribution of the number of attacks and the number of injured patients showed a positive correlation.

### Demographic and temporal characteristics

The basic demographic and temporal characteristics of the two injury mechanisms are demonstrated in Table 1. The injury rate per incident was significantly higher in injuries caused by explosions compared to the firearm mechanism (9.09 vs. 5.6, χ^2^ *P* = 0.0009). Most of the patients (n = 1974, 81%) were male, while the remaining 19% of the cases were female. Firearm injuries included a significantly higher proportion of men compared to explosion injuries. While firearm injuries predominantly in young patients between the ages of 15–44, there was no significant difference between age groups in explosion injuries. The explosion injuries occurred almost exclusively on weekend days, while firearm injuries mostly occurred after sunset and night (χ^2^ *P* < 0.005).

**Table 1:** Circumstantial characteristics, age, and sex distribution of terrorism-related injury mechanisms.

	Explosion, N = 1073, No. (% 44)		Firearm, N = 1.353, No. (% 56)	
	N	%	N	%
Number of attacks	118	33	241	67
Injury rate by mechanism*	9.09		5.6	
**Day of week**				
Weekday	186	8	1194	77
Weekend**	887	92	159	23
**Time of day**				
6 am–1:59 pm	319	30	198	14
2 pm–9:59 pm	588	55	412	31
10 pm–5:59 am	166	15	743	55
**Sex**				
Men	751	70	1223	90
Women	322	30	130	10
**Age, year**				
15–29	201	19	492	36
30–44	286	26	513	38
45–59	317	30	214	16
≥ 60	269	25	134	10

*Number of injured patients/number of attacks.

**In Somali, the weekend is on Thursday and Friday.

### Injury features

**Figure 2:**
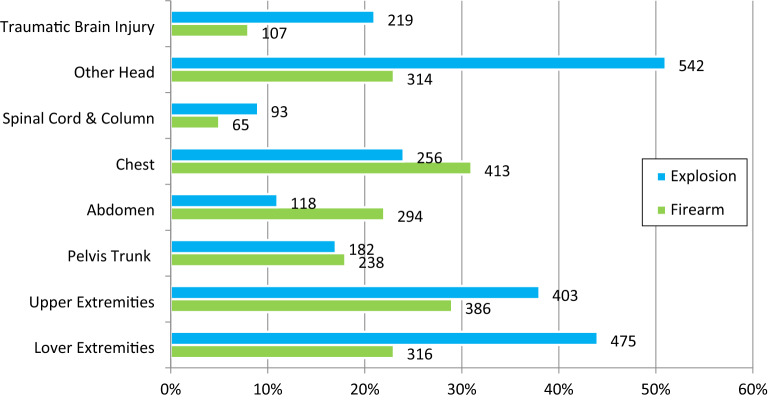
Injured body area by injury mechanism.

**Figure 3:**
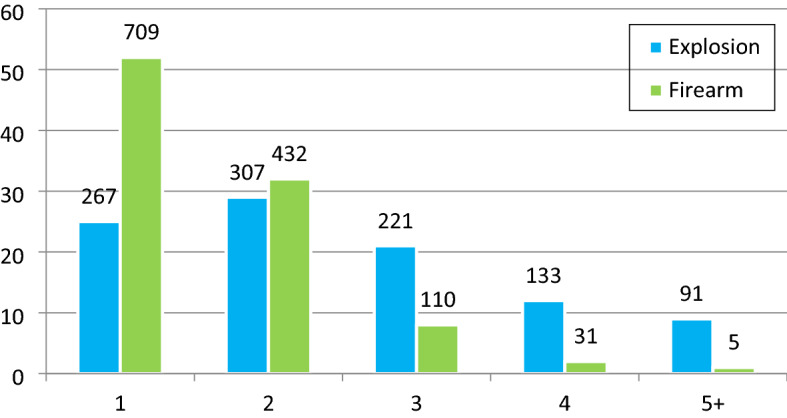
Number of body regions injured by injury mechanism (based on nine body regions).

The chest, abdomen, and pelvis were the most common injury sites in firearm wounds, but all other body parts were injured more frequently in explosions (Fig. 2). Head, brain, and lower extremity injuries frequently occurred in the explosions. Spinal cord and spine injuries occurred at a lower rate. Multiple body site injuries occurred more frequently in explosion injuries (75%) than firearm wounds (48%) (χ^2^ *P* < 0.001). Figure 3 shows the numbers of relevant body parts injured by explosion and firearm mechanisms. Internal injuries were divided into three body regions (head, chest, and abdomen) and their combinations for analysis purposes. 40.8% (N = 992) of the patients suffered internal injuries, of which 502 were explosion injuries, and 490 were firearm injuries (Table 2). The frequency of internal injuries in explosion injuries was higher than in firearm wounds (46.7% vs. 36.2%). Single chest injuries were common in firearm injuries, while only head injuries were more common in explosion patients. Injuries affecting the chest and abdomen occurred in 21% of firearm wounds vs. 9% of explosion victims. In patients with internal injuries, other multisite injuries were more common in explosion injuries (11.2 vs. 1.4% per explosion). The mortality rate in patients with internal injuries was higher in firearm wounds (57.1% vs. 42.9%). Among the explosion injuries, those with multisite injuries and those with firearm injuries resulted in more deaths due to head injuries. The severity score and inpatient mortality rates for injured body parts are summarized in Table 2.

**Table 2:** Distribution of internal injuries according to injury mechanism by body regions and their effects.

	Total		ISS 25 +	Dead patient
	N	%	%	%
Total	992	100	52.3	25.6
Explosion (total)	502	50.6	47.1	42.9
Head	173	34.5	21.6	12.3
Chest	144	28.7	46.5	14.8
Abdomen	82	16.3	23.8	10.6
Chest and abdomen	47	9.3	79.8	24.5
Head and other*	56	11.2	93.4	37.8
Firearm (total)	490	49.4	52.9	57.1
Head	69	14.1	29.6	34.4
Chest	184	37.6	25.4	21.3
Abdomen	126	25.7	41.3	9.5
Chest and abdomen	104	21.2	59.7	13.3
Head and other*	7	1.4	–	21.5

*Head and other include: head and chest, head and abdomen and head, and both chest and abdomen.

*ISS* Injury severity score.

One hundred seventy-three patients had burn injuries (7.1%), and most (N = 141, 81.5%) were explosion injury victims. The rate of open wounds was significantly higher in patients with firearm wounds (81% vs. 34%, χ^2^ *P* = 0.0009), but fractures (33% vs. 48%, χ^2^ *P* = 0.0005) were lower.

Explosion attacks were carried out with two sub-groups of mechanisms: live bombs and bombs placed on vehicles. With a large majority (N = 71, 60.2%), suicide bombings were more frequent. While the number of patients injured and killed in suicide bombings was significantly higher, the severity of the injury and the rate of internal injuries were lower. The distribution of internal injury, injury severity, and death rate according to the two subgroup mechanisms of explosion injuries is summarized in Table 3.

**Table 3:** Characteristics of explosion injuries according to 2 different subgroup mechanisms.

	Number of attacks		Number of injured patients		Internal injury		ISS 25 +	Number of dead patient
	N	%	N	%	N	%	%	%
Live bomb	71	60.2	802	74.7	219	43.6	41.6	60.6
Bombs placed on vehicles	47	39.8	271	25.3	283	56.4	58.4	39.4

*ISS* Injury severity score.

### Injury severity

Table 4 shows the distribution of severity groups for both injury mechanisms. Patients injured due to the explosion have a greater rate of severe and critical injuries than those injured by firearms (30.2% vs. 21.2%, χ^2^ *P* < 0.001). More than 52% of the patients with internal injuries had a critical or fatal injury (with an ISS over 25). The severity of the injury was higher in patients with internal injuries, and 70% of patients suffered from a serious (ISS16 +) injury. 93.4% of explosion victims with head and other injuries and 79.8% of patients with explosion -related chest and abdominal injuries had an ISS of 25 or higher.

**Table 4:** Distribution of injury severity score by injury mechanism, nature of injury and number of injured body areas.

	Injury severity score (ISS) group			
	1–8	9–14	16–24	25–75
**Injury mechanism**				
Explosion	55.6	12.1	9.1	23.2
Firearm	49.3	18.8	11.4	20.5
**Type of injury**				
Internal injury	11.6	17.6	18.5	52.3
Other	61.2	20.7	9.8	8.3
**Number of injured body parts**				
1	68.9	18.6	9.3	3.3
2	43.2	21.9	19.8	15.2
3	25.4	20.7	13.7	40.3
4	17.5	18.3	21.5	42.8
5 and above	4.3	6.9	27.8	61.1

The sum of the percentages in each row is 100%. Body region groups are defined as: TBI, other head, VCI/SCI, chest, abdomen, pelvis, back and buttock, upper extremities, lower extremities and other.

### Utilization of hospital resources

A total of 582 patients (24%) were hospitalized in the intensive care unit (ICU) (Table 5). The rate of patients requiring ICU treatment was higher in explosion injuries than in firearm victims, but no significant difference was found (25.2% vs. 22.9%). There was no difference between the two groups regarding total hospital stay. Multidimensional injuries such as burns and penetrating injuries and patients with multiple body site injuries had higher rates of intensive care unit stay. A surgical procedure was performed in 1319 patients (54.3%). Surgical applied firearm wounds were significantly higher in explosion injuries than firearm victims (62.3% vs. 44.3%).

**Table 5:** Characteristics of injury, death, and hospitalization according to injury mechanism.

	Explosion		Firearm	
	N	%	N	%
Intensive care unit stay*	271	25.2	311	22.9
Median (IQR)†	4 (2–8)		3 (1–6)	
**Length of stay‡**				
1–6 days	614	57.2	770	56.9
7–14 days	229	21.3	298	22.0
15 and above	207	19.2	257	18.9
Median	4 (1–10)		4 (1–11)	
Inpatient death**				
Death	132	12.3	148	10.9
**Inpatient death time**				
Within 1 day	83	62.9	129	87.1
2–7 days	31	23.5	13	8.9
8 and above	18	13.6	6	4.0
Surgical procedures§	476	44.3	843	62.3

*χ^2^ tests for *P* value = 0.614.

†Wilcoxon test for *P* value = 0.0718.

‡χ^2^ tests for *P* value = 0.4218.

**χ^2^ tests for *P* value = 0.116.

§χ^2^ tests for *P* value = 0.0652.

### Mortality rate and time

**Figure 4:**
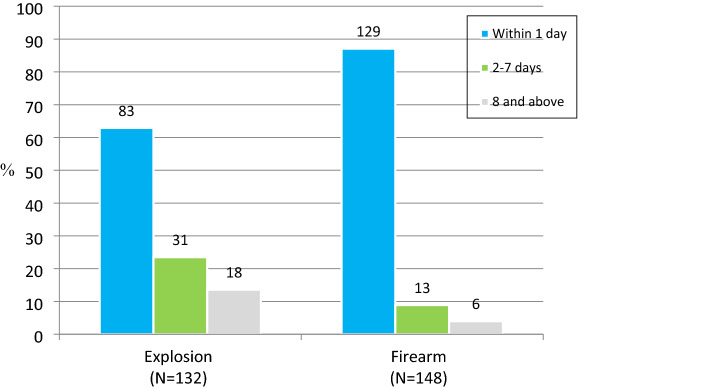
Time of Inpatient Death Due to Injury (Patients who died in hospital only) %, is the percentage in each cause population.

The inpatient mortality rate were 11.6% (n = 280 patients) (Table 5). There was no statistically significant difference in mortality rate between the groups (χ^2^ *P* = 0.11); However, a significantly larger proportion of firearm victims died within the first day of hospital admission (87%, *P* < 0.0001), while explosion injuries were more likely to occur later (Fig. 4). Outcomes for internal injury patients were significantly worse, with a mortality rate of 25.6% (Table 2). Inpatient mortality rates were higher in patients with multidimensional injuries in more than one body region.

## Discussion

This study focused on patients who were injured and hospitalized over a 7-year after terrorist attacks in Somalia. In recent years, almost all terrorism-related injuries in Somalia have been caused by firearm and explosion injuries. Understanding injuries, anatomical injury patterns, and their impact on survival are complex11. The effects of terrorist injuries on victims and the differences between injury patterns caused by the two different mechanisms were reviewed to increase the knowledge and skill level of health personnel, and raise awareness about the approach to these injuries and traumas that frequently occur in Somalia.

Explosion victims generally come in mass casualty or at short intervals and cause a sudden patient load that can affect patient care, while firearm injuries occur individually over a wider period and cause a less patient burden. In a mass injury scenario, "minimum acceptable care" must be provided^4^.

The literature has reported that terror-related explosion-related injury mechanisms reveal different injury patterns. The initial explosion can cause tissue damage, and pushed pieces can cause penetrating injuries. Impacts from throwing the victim's body against a fixed object usually cause blunt injuries but can also cause penetrating injuries. Additionally, victims often suffer burns from the heat emitted by the explosive device or the fire ignited by the explosion^4,11^. Explosion injuries in Somalia revealed a previously unknown form of injury created by new types of shrapnel, such as explosives or other sharp metal objects from vehicles in which explosives were planted. Our current study revealed that these metals cause penetrating injuries that are often difficult to detect at the initial trauma view. Therefore, even victims who come to the hospital with minor trauma at first glance may require detailed secondary trauma examination and diagnostic investigations. Combinations of mass injuries originating from different mechanisms have been described in the literature^12,13^.

The large proportion (30%) of internal penetrating and blunt injuries exhibited in this study is a significant cause of the high rate of serious injury in terror-related traumas compared to other forms of trauma^14,15^. The most frequently injured body regions in this study were the head and extremities, similar to prospective, longitudinal analysis of injuries by a U.S. Army Brigade Combat Team during "The Surge" phase of Operation Iraqi Freedom that reported the distribution of injuries as follows: head/neck 36.2%, thorax 7.5%, abdomen 6.9%, and extremities 49.4%^16^. Internal injuries were predominant to the head and chest (63%) in explosion injuries and chest and abdominal injuries (63%) in firearm injuries, but the overall rate was similar.

For death from trauma to be preventable, the underlying injury burden must first be assessed and considered survivable or potentially survivable. Determination of injury-free survival has traditionally been based on subject expert judgment, physiological injury severity, and anatomical injury severity^17^. The study shows that firearm wounds are mostly isolated to specific body regions, while explosion injuries involve multiple anatomical body regions. The authors acknowledged that multiple body site injuries significantly cause increased overall injury severity. Similar to the results from Israel, this study reported that the rate of severe (ISS 16 +) injury in patients with five or more injuries is almost 89%, which is seven times higher than in patients with one body area injury^15^. By definition, ISS will likely increase with multiple body area injuries. However, the distribution of injury severity is noteworthy, as patients injured in explosion s have a large proportion of minor injuries versus a smaller proportion of critical fatal injuries, as noted in the present study. Explosion injuries may underestimate the severity of injury due to a larger proportion of minor injuries with a smaller proportion of critical fatal injuries, besides the multiple body region injuries at a higher rate of about 75% compared to firearm wounds that include one body region injury at a high rate of 52%^18^. Similar to our findings, A retrospective study of 1784 civilians and 802 soldiers reported that critical injuries and injuries to multiple body regions were more likely in terror than in war^19^. These results demonstrate the importance of injury mechanisms in understanding anatomical injury patterns^20,21^. This finding warrants the need to create a new ISS which will account for multidimensional injuries.

Injuries from explosive mechanisms involve more than one body region, whereas firearm wounds are more isolated to specific body regions such as the chest and abdomen, as seen in our study population. The study's findings are consistent with analyzes of more than 1,000 terrorism-related injuries from explosive and firearm mechanisms reported from the Israel National Trauma Registry and more than 5000 from the U.S. Department of Defense Trauma Registry (DoDTR)^22,23^.

The length of hospitalization and mean stay in the intensive care unit of both groups were close. This finding has significant implications for hospital resource use, as intensive care beds are a limited resource in low-income countries. The inpatient mortality rate was 11.6%, which coheres with the previously reported studies^24^. There was no statistically significant difference in mortality rate between the groups. However, a significantly larger proportion of firearm victims died within the first day of hospital admission, while explosion injuries were more likely to occur later. The probable reason for this may be that the explosion attacks occur in the city close to the health facilities, and the transfer time is short, while the firearm attacks occur outside the city. Tailored protocols for traumatized patient assessment, management algorithm, and initial treatment should differ between firearm and explosion victims. Understanding injury patterns enables the development of trauma care systems and the corresponding reduction in deaths.

## Strengths and limitations

The present is the first and only comprehensive study examining terrorist acts and their effects reported from Somalia. Increasing studies like this will help to raise awareness against international terrorism, which has become a global problem and needs to be solved with the common attitude of all countries. During the study period, the total number of people injured or killed by terrorist incidents throughout the country could not be reached.

## Conclusion

The findings of the study revealed that suicide bombings explosions are associated with an increased injury rate per incident, multiple body site injuries, a greater rate of severe and critical internal injuries, and a higher mortality rate. The inpatient mortality rate was 11.6% of the cases. There was no statistically significant difference in mortality rate between the groups. However, a significantly larger proportion of firearm victims died within the first day of hospital admission, while explosion injuries were more likely to occur later.

## Abbreviations

AIS: Abbreviated injury scale
DoDTR: US Department of Defense Trauma Registry
ISS: Injury severity score
ICU: Intensive care unit
HIS: Hospital information system
LOS: Length of stay
TBI: Traumatic brain injury
